# Roles of lncRNA LVBU in regulating urea cycle/polyamine synthesis axis to promote colorectal carcinoma progression

**DOI:** 10.1038/s41388-022-02413-8

**Published:** 2022-07-29

**Authors:** Xiangqi Meng, Jingxuan Peng, Xiaoshan Xie, Fenghai Yu, Wencong Wang, Qihao Pan, Huilin Jin, Xiaoling Huang, Hongyan Yu, Shengrong Li, Dianying Feng, Qingxin Liu, Lekun Fang, Mong-Hong Lee

**Affiliations:** 1 Guangdong Provincial Key laboratory of Colorectal and Pelvic Floor Disease, The Sixth Affiliated Hospital, Sun Yat-sen University, Guangzhou, 510655 China; 2 Guangdong Research Institute of Gastroenterology, The Sixth Affiliated Hospital, Sun Yat-sen University, Guangzhou, 510655 China; 3 Department of Clinical Biological Resource Bank, Guangzhou Institute of Pediatrics, Guangzhou Women and Children’s Medical Center, Guangzhou Medical University, Guangzhou, 510623 China; 4 Pharmacy College, Chengdu University of Traditional Chinese Medicine, Chengdu, 611137 China; 5 Department of Colorectal Surgery, The Sixth Affiliated Hospital, Sun Yat-sen University, Guangzhou, 510655 China

**Keywords:** Gastrointestinal cancer, Non-coding RNAs

## Abstract

Altered expression of Urea Cycle (UC) enzymes occurs in many tumors, resulting a metabolic hallmark termed as UC dysregulation. Polyamines are synthesized from ornithine, and polyamine synthetic genes are elevated in various tumors. However, the underlying deregulations of UC/ polyamine synthesis in cancer remain elusive. Here, we characterized a hypoxia-induced lncRNA LVBU (lncRNA regulation via BCL6/urea cycle) that is highly expressed in colorectal cancer (CRC) and correlates with poor cancer prognosis. Increased LVBU expression promoted CRC cells proliferation, foci formation and tumorigenesis. Further, LVBU regulates urea cycle and polyamine synthesis through BCL6, a negative regulator of p53. Mechanistically, overexpression of LVBU competitively bound miR-10a/miR-34c to protect BCL6 from miR-10a/34c-mediated degradation, which in turn allows BCL6 to block p53-mediated suppression of genes (arginase1 *ARG1*, ornithine transcarbamylase *OTC*, ornithine decarboxylase 1 *ODC1*) involved in UC/polyamine synthesis. Significantly, ODC1 inhibitor attenuated the growth of patient derived xenografts (PDX) that sustain high LVBU levels. Taken together, elevated LVBU can regulate BCL6-p53 signaling axis for systemic UC/polyamine synthesis reprogramming and confers a predilection toward CRC development. Our data demonstrates that further drug development and clinical evaluation of inhibiting UC/polyamine synthesis are warranted for CRC patients with high expression of LVBU.

## Introduction

Colorectal cancer (CRC) is one of the deadliest cancer types with different molecular phenotypes/strong resistance to therapies [1] and is a cancer type with a very high mortality rate [2]. Thus, it is important to identify potential cancer biomarkers for effective treatment strategies for CRC [3, 4]. Deregulations of long non coding RNAs (lncRNAs) are involved in cancer through diverse physiological and pathophysiological functions [5, 6]. However, the pathophysiological contribution of lncRNAs aberrations in CRC has not been fully characterized.

Hypoxia is known to promote cancer formation and cause treatment resistance [7]. Subregions of tumors usually have low levels of oxygen (hypoxia). These hypoxic areas elicit potential risks of promoting genomic instability or facilitating distant metastasis based on studies of more than 8000 tumor samples [8]. Hypoxia-inducible factor (HIF) is responsible for regulating metabolic reprogramming [9], innate immunity, such as immunity and inflammation [10, 11], and the unfolded protein response pathways [12]. Although it has been reported that hypoxia can also promote cancer progression through regulating some lncRNAs [13], many molecular cancer characteristics of lncRNAs due to tumor hypoxia remain to be further studied.

The urea cycle (UC) is a pathway to dispose systemic nitrogen waste derived from the breakdown of nitrogen-containing metabolites (such as ammonia and glutamine) into urea [14]. UC dysregulation (UCD) is a deregulated metabolic phenomenon for cancer, which is caused by specific alterations of UC enzymes [15]. For example, p53 suppresses ureagenesis and elimination of ammonia, thereby inhibiting tumor growth [16]. When p53 is lost, deregulated ureagenesis leads to pyrimidine synthesis, thereby facilitating cancer growth [17]. UCD affects carcinogenesis, mutagenesis, and immunotherapy response; therefore, characterizing the molecular regulations of UCD in tumorigenesis is critical for diagnosis and therapy [18]. Further, Ornithine decarboxylase 1 (*ODC1*) is a critical enzyme using ornithine from urea cycle for polyamine biosynthesis, and is frequently deregulated in cancer [19]. Enhanced polyamine biosynthesis facilitates cancer growth [20]. However, the contribution of *ODC1* deregulation in CRC remains not well characterized.

In this study, we demonstrate that the frequent overexpression of LVBU results in the UC reprogramming/polyamine synthesis phenotype in CRC via regulating BCL6 and p53, which in turn promotes tumor growth/cell cycle proliferation, foci formation, tumorigenicity, and correlates with poor cancer survival. Our study uncovers LVBU as an important cancer biomarker regulating UC/polyamine synthesis pathway and provides insight into potential cancer treatment strategies.

## Results

### Hypoxia-induced lncRNA-LVBU is upregulated in colorectal cancer and correlated with poor prognosis

**Fig. 1:**
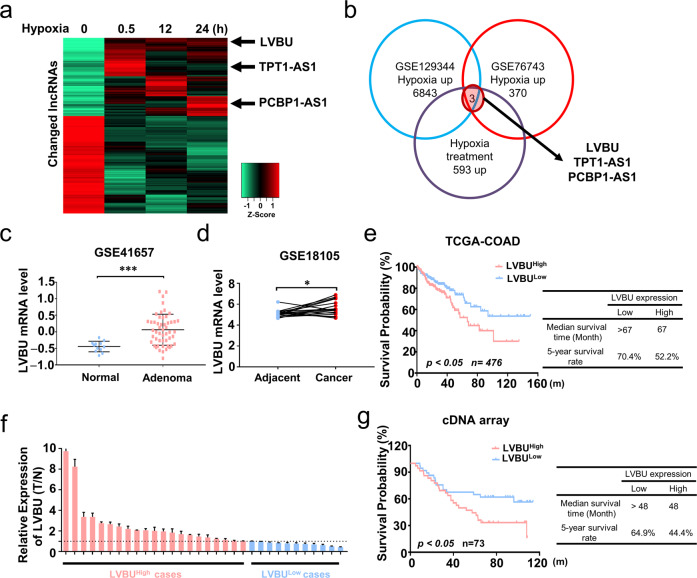
LVBU is upregulated in colorectal cancer and correlated with poor prognosis. **a** HCT116 cells were cultured under hypoxia condition (1% O_2_) for up to 24 h. At the indicated time points, cells were harvested, and RNA was isolated to determine lncRNA expression levels by high throughput sequencing. The heatmap depicts lncRNAs for which a > 1.5-fold change in expression was observed in all time points, compared to time zero. Note that each row represents an individual lncRNA, and the fold change is log2 transformed. **b** Overlapping upregulated lncRNAs under hypoxia condition among high throughput sequencing results and GEO datasets (GSE129344 and GSE76743). **c** Expression level of LVBU in normal mucosa and adenoma tissues (GSE41657). Unpaired student’s *t* test was performed (***p < 0.001). **d** Expression level of LVBU in adjacent nontumor tissue and cancer samples. (GSE18105). Paired student’s *t* test was performed (*p < 0.05). **e** Kaplan–Meier overall survival (OS) curves based on LVBU expression in colon cancer tissues (COAD) from TCGA database. The median expression value (COAD) was used to define the high and low expression group, and log-rank analysis was used to test for significance. **f** Relative LVBU expression levels in 32 paired samples of colorectal cancer and adjacent nontumor tissues measured by qRT-PCR (paired student’s *t* test, **p < 0.01). **g** Kaplan–Meier overall survival (OS) curves based on LVBU expression in colon cancer tissues (COAD) from cDNA array (Excluding mucinous cancer type). The median expression value was used to define the high and low expression group, and log-rank analysis was used to test for significance.

In this study, we aimed to explore lncRNAs regulated under hypoxia condition and investigate their potential roles and mechanisms during CRC development. Transcriptional responses of lncRNAs in HCT116 cells were assessed under normoxia or increasing time intervals of hypoxia condition (0.5, 12 and 24 h). There were 593 upregulated lncRNAs, while 740 downregulated lncRNAs (Fig. 1a). After overlapping the upregulated lncRNAs with published GEO database, 3 potential candidate lncRNAs were found (Fig. 1b). Among these, LINC00205 (ENSG00000223768), which we named lncRNA-LVBU, is encoded by a DNA sequence located at chromosome 21. Through bioinformatic analysis of GEO database, we found LVBU was upregulated in colorectal adenoma and cancer tissues (Fig. 1c, d). Moreover, Kaplan–Meier analysis showed that colon cancer patients bearing high-LVBU expression displayed a significantly shorter median survival time (67 months) and lower 5-year survival rate (52.2%) when compared with those of low-LVBU expression (TCGA COAD dataset) (Fig. 1e). To further validate these findings, quantitative RT-PCR analysis of LVBU expression in 32 paired samples of CRC tissue and adjacent nontumor mucosa revealed that LVBU expression was significantly upregulated in cancer tissues (Fig. 1f). Further, our CRC patients’ cDNA microarray analysis also demonstrated that higher expression of LVBU correlated with poor overall survival in colorectal adenocarcinoma tissues (Fig. 1g). Associations of the expression of LVBU and CRC patients’ clinicopathological information were listed in Table S1. Thus, we demonstrated that the expression of LVBU was upregulated by hypoxia and correlated with poor prognosis in CRC.

### HIF-1α stimulated LVBU transcription under hypoxia condition

**Fig. 2:**
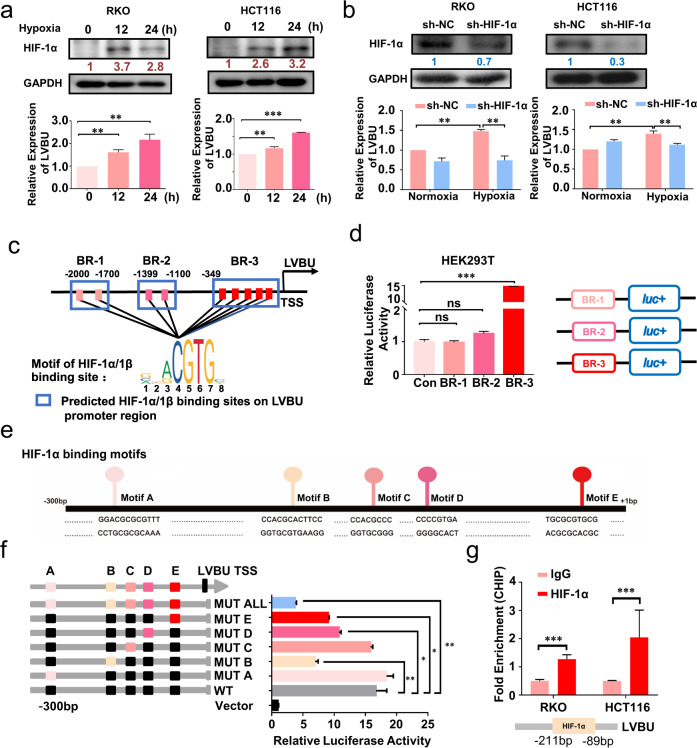
HIF-1α binds to LVBU promoter to promote gene transcription of LVBU. **a** Upregulation of LVBU in RKO and HCT116 cells under hypoxia condition (1% O_2_) was detected by qRT-PCR. HIF-1α expression was detected by western blot at the indicated time points after hypoxia treatment (*N* = 3 independent samples, **p < 0.01; ***p < 0.001). **b** The expression of LVBU was detected by qRT-PCR under normoxia or hypoxia condition when HIF-1α was knocked down (*N* = 3 independent samples, **p < 0.01). **c** Predicted binding regions (BRs) of HIF-1 α/1β at the promoter (−2000 bp–TSS) of LVBU were shown. The prediction was performed with the website JASPAR. **d** Luciferase activities were detected by dual luciferase reporter assay after HEK-293T cells transfected with the indicated reporter plasmids under hypoxia condition (1% O_2_). *N* = 3 independent samples, ***p < 0.001. **e** Predicted binding motifs of HIF-1α at the promoter region (−300 bp- +1 bp) of LVBU were shown. **f** Schematic drawing of constructs of LVBU promoter inserted into pGL3-basic luciferase reporter plasmid. Luciferase activity was detected by dual luciferase reporter assay after HEK-293T cells transfected with the indicated reporter plasmids (*N* = 3 independent samples, *p < 0.05; **p < 0.01). **g** Chromatin immunoprecipitation (ChIP) assays confirmed the binding site of HIF-1α on LVBU promoter in RKO and HCT116 cells (*N* = 3 independent samples, ***p < 0.001).

Using the 5′ and 3′ rapids amplification of cDNA ends (RACE) assay, we discovered that lncRNA-LVBU is a 4474-nt transcript and consists of four exons and three introns (Fig. S1a–c). Furthermore, subcellular fractionation analysis showed that LVBU was distributed in both cytoplasm and nucleus (Fig. S2). In order to understand the mechanism of LVBU upregulation under hypoxia condition, we hypothesized that HIF1 may have an impact and found that expression of LVBU was upregulated under hypoxia condition in a HIF-1α-dependent manner (Fig. 2a, b). We further performed bioinformatic analysis of LVBU promoter region using JASPAR website. Nine potential binding sites of HIF-1α were found (within 3 binding regions (BR), Fig. 2c). Luciferase reporter assays indicated that only binding region 3 (BR-3) could be induced under hypoxia condition (Fig. 2d). Next, the individual predicted binding sites (Motifs A-E) within BR-3 were further mutated respectively, and luciferase reporter assays indicated that Motif B, D, and E were the potential binding sites of HIF-1α (Fig. 2e, f). Further mutation all of these sites abolished the induction by hypoxia condition (Fig. 2f). Finally, chromatin immunoprecipitation (CHIP) assays confirmed that HIF-1α binds to the promoter of LVBU within -211— -89 bp region (Fig. 2g). These findings indicate that the upregulation of LVBU is mediated by the binding of HIF-1α to LVBU promoter in CRC cells.

### LVBU promoted CRC cell proliferation in vitro

Since LVBU was expressed under hypoxia condition and aberrantly upregulated in CRC, we next characterized its biological roles. Knockdown expression of LVBU with either siRNA or antisense oligonucleotide (ASO) remarkably inhibited CRC cells proliferation and foci formation (Fig. S3a, b). We further constructed LVBU overexpression plasmids, and found that overexpression of LVBU significantly increased cells proliferation and foci formation (Fig. S3c). Furthermore, we also found that knockdown expression of LVBU could significantly induce cell senescence (SA-β gal staining) under starvation condition and cause G1 cell cycle arrest in CRC cells (Fig. S3d, e).

### LVBU regulated CRC cell proliferation through p53/nitrogen metabolism pathway

**Fig. 3:**
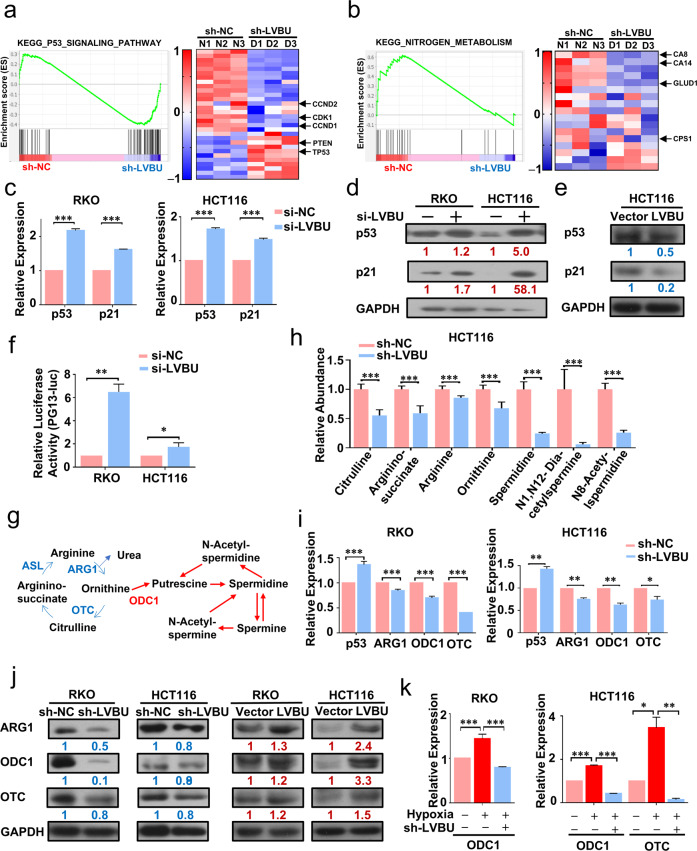
LVBU regulates CRC cell proliferation through impacting p53 axis and urea cycle. **a**, **b** GSEA results show p53 signaling pathway upregulated and nitrogen metabolism pathway downregulated in sh-LVBU group. sh-NC, negative control. **c** p53 and p21 mRNA expression levels were detected by qPCR after knockdown of LVBU expression with siRNA (*N* = 3 independent samples, ***p < 0.001). **d**, **e** p53 and p21 protein levels were detected by western blot after knockdown LVBU with siRNA or overexpression of LVBU in HCT116 or RKO cells. **f** HCT116 and RKO cells were transfected with p53 reporter (PG13-luc) and renilla plasmids. Relative luciferase activity was detected after cells transfected with either si-NC or si-LVBU (*N* = 3 independent samples, *p < 0.05; **p < 0.01). **g** Schematic depicting the metabolites and key enzymes from urea cycle and polyamine pathways. Blue and red arrows indicate urea cycle and polyamine metabolic pathways respectively. **h** Urea cycle/ polyamine metabolic pathway related metabolites were detected by LC-MS after LVBU knockdown with shRNA (*N* = 6 independent samples, ***p < 0.001). **i** p53, ARG1, ODC1 and OTC mRNA expression levels were detected by qRT-PCR after LVBU knockdown with shRNA in RKO and HCT116 (*N* = 3 independent samples, *p < 0.05; **p < 0.01; ***p < 0.001). **j** ARG1, ODC1 and OTC protein levels were detected by western blot after knockdown LVBU with shRNA or overexpression of LVBU in HCT116 and RKO cells. **k** ODC1 and OTC mRNA expression were detected after hypoxia and shLVBU treatment in HCT116 and RKO cells (*N* = 3 independent samples, *p < 0.05; **p < 0.01; ***p < 0.001).

To identify the mechanism of LVBU involved in CRC progression, high throughput sequencing analysis was performed after LVBU was knocked down with shRNA. Gene set enrichment analysis demonstrated that p53 signaling pathway related genes were particularly enriched in LVBU knockdown group, such as p53 and PTEN (Fig. 3a). On the other hand, nitrogen metabolism pathway related genes were downregulated in LVBU knockdown group, such as CPS1 and GLUD1 (Fig. 3b). These data suggested that LVBU might regulate CRC cell proliferation through p53/nitrogen metabolism pathway. To test this hypothesis, we first examined the impact of LVBU on p53 pathway. Knockdown the expression of LVBU leads to significant upregulation of both the mRNA and protein levels of p53 and p21 (Fig. 3c, d). Congruently, overexpression of LVBU could decrease both p53 and p21 expression (Fig. 3e). p53 luciferase reporter assay showed that knockdown LVBU expression could significantly increase luciferase activity in CRC cells (Fig. 3f).

To investigate the impact of LVBU on nitrogen metabolism, we performed LC-MS metabolite analysis after LVBU knockdown and showed that the intermediate metabolites of urea cycle (citrulline, arginino-succinate, arginine, ornithine) and polyamine synthesis pathway (spermidine, N1, N12 diacetylacetylspermidine, N8-acetylspermidine) were decreased in LVBU knockdown group (Fig. 3g, h), indicating LVBU’s role in regulating urea cycle/polyamine synthesis. Urea cycle and polyamine synthesis pathways play important roles in cancer development. It is important to point out that Kaplan–Meier analysis showed that high expression of two enzymes, ornithine transcarbamoylase (OTC) and argininosuccinate lyase (ASL), was correlated with poor survival in rectal carcinoma patients (Fig. S4a, b). It is possible that LVBU in CRC may have impact on one of these regulators to regulate urea cycle. Indeed, LVBU knockdown leads to downregulation of ARG1 and OTC (Fig. 3i, Fig. S4c). These two genes happened to be suppressed by p53. We then identified that LVBU knockdown-mediated p53 upregulation led to the downregulation of ARG1 and OTC (Fig. 3I, j), suggesting a LVBU-p53 link. Congruently, overexpression of LVBU increased the protein expression of those genes (Fig. 3j). Interestingly, ornithine decarboxylase 1(ODC1), which is also suppressed by p53 and critical in polyamine synthesis, was also downregulated in response to LVBU knockdown (Fig. 3i), and upregulated when LVBU was overexpressed (Fig. 3j). These data suggest that LVBU can positively regulate the expression of ARG1, OTC and ODC1 through repressing p53 expression.

Interestingly, our KEGG enrichment analysis showed that nitrogen metabolism, carbon metabolism, and HIF1-signaling pathways are within the top 30 KEGG enriched pathways at the 12 and 24 h hypoxia time points (Fig. S5a), implying the connection among these three pathways. Indeed, qPCR experiment results showed that hypoxia condition leads to the elevation of ODC1 and OTC in CRC cells (Fig. S5b). Importantly, this elevation was reversed by LVBU knockdown (Fig. 3k), illustrating the axis of hypoxia-LVBU in regulation nitrogen metabolism. Taken together, these results indicated that hypoxia-induced LVBU regulates p53 and urea cycle/polyamine synthesis pathway.

### LVBU-BCL6 axis negatively regulates p53, thereby promoting urea cycle

**Fig. 4:**
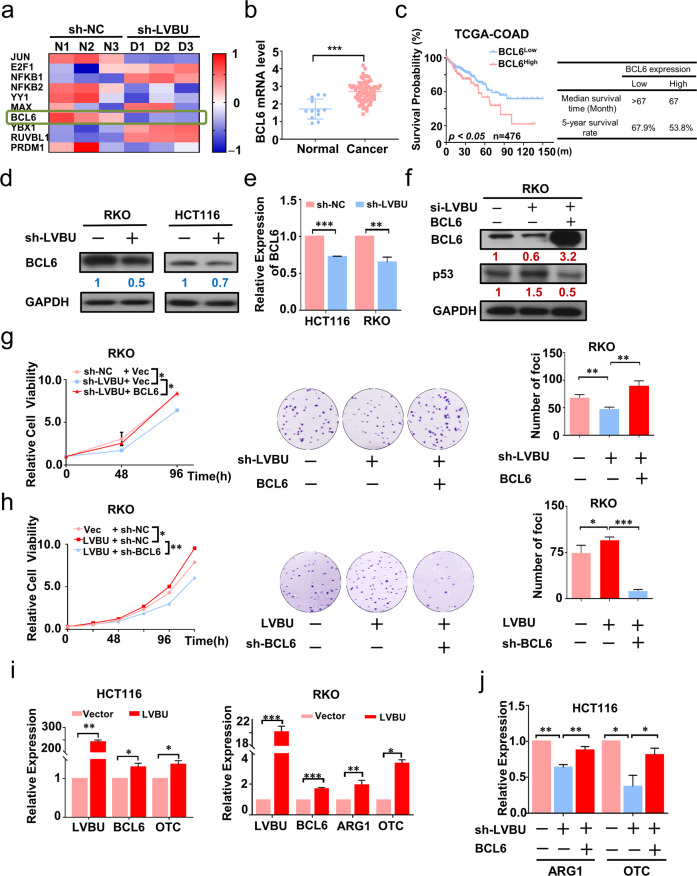
BCL6 is the downstream target of LVBU to regulate p53/UC pathway. **a** Expression of potential p53 transcriptional regulators after LVBU knockdown. **b** Expression level of BCL6 is high in cancer samples (Oncomine, Hong Colorectal). Student’s *t* test was used (***p < 0.001). **c** Kaplan–Meier overall survival (OS) curves based on BCL6 expression in colon cancer tissues (COAD) from TCGA database. ROC curve was used to define the high and low expression group, and log-rank analysis was used to test for significance. **d**, **e** Expression of BCL6 was determined by qRT-PCR and western blot after LVBU knockdown in HCT116 and RKO cells (*N* = 3 independent samples, **p < 0.01; ***p < 0.001). **f** Immunoblot analysis of BCL6 and p53 expression after cells transfected with the indicated plasmids and siRNAs. **g**, **h** Proliferation rate and foci formation of RKO cells were measured after transfecting with the indicated constructs (*N* = 3 independent experiments, *p < 0.05; **p < 0.01; ***p < 0.001). **i** BCL6, OTC and ARG1 mRNA expression in HCT116 and RKO cells were determined by qRT-PCR after transfecting with LVBU overexpression plasmid (*N* = 3 independent samples, *p < 0.05; **p < 0.01; ***p < 0.001). **j** Cells induced with or without Dox for LVBU knockdown were transfected with the indicated constructs. ARG1 and OTC mRNA expression was determined by qRT-PCR (*N* = 3 independent samples, *p < 0.05; ** *p* < 0.01).

In order to further investigate the mechanism behind LVBU-mediated down regulation of p53, we examined the mRNA stability of p53 under actinomycin D treatment and found that knockdown of LVBU could not affect p53 mRNA turnover rate (Fig. S5c). Next, we searched for several potential p53 transcriptional factors regulated by LVBU, and found that BCL6 [21, 22], which is a transcriptional repressor of p53 [23], was downregulated when LVBU was knocked down (Fig. 4a). Significantly, BCL6 was also upregulated in CRC tumor samples, and high expression of BCL6 was correlated with poor prognosis of colon cancer patients based on TCGA datasets analysis (Fig. 4b, c). LVBU knockdown leads to downregulation of BCL6 in CRC cells (Fig. 4d, e), suggesting a regulatory relationship between LVBU and BCL6. Most importantly, LVBU knockdown-mediated p53 upregulation could be reversed by BCL6 overexpression (Fig. 4f). Therefore, a LVBU-BCL6-p53 regulatory axis can be established. Congruently, functional studies demonstrated that overexpression of BCL6 can reverse LVBU knockdown-mediated growth inhibition as determined by both CCK8 and foci formation assays (Fig. 4g). On the other hand, LVBU-mediated growth promotion can be mitigated by knockdown of BCL6 (Fig. 4h). Interestingly, LVBU overexpression leads to concurrent elevation of BCL6, OTC, and ARG1 (Fig. 4i). On the other hand, LVBU knockdown caused downregulation of ARG1/OTC gene expression, which can be reversed by expression of BCL6 (Fig. 4j), suggesting that BCL6 is indeed linked to LVBU-mediated nitrogen metabolism. Taken together, these data indicated that LVBU regulates BCL6, which in turn suppresses p53, and subsequently upregulates the expression of ARG1 and OTC.

### LVBU regulates BCL6 expression through sponging miR-10a/miR-34c

**Fig. 5:**
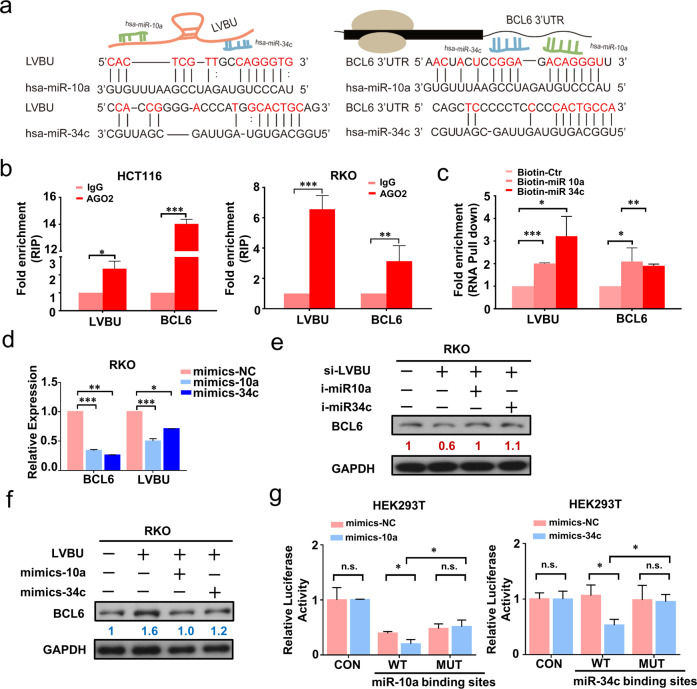
LVBU upregulates BCL6 expression through sequestering miR-10a/34c. **a** Schematic drawing of miR-10a/34c binding sites on LVBU and BCL6 3’UTR. The binding sites were predicted by bioinformatic website DIANA. **b** RIP experiments were performed with the AGO2 antibody in HCT116 and RKO cells. Specific primers were used to detect the enrichment of LVBU and BCL6 by qRT-PCR (*N* = 3 independent experiments, *p < 0.05; **p < 0.01; ***p < 0.001). **c** RNA pull down assays were performed by using biotin-labeled miR-10a and miR-34c. LVBU and BCL6 mRNA level were detected by qRT-PCR (*N* = 3 independent experiments, *p < 0.05; **p < 0.01; ***p < 0.001). **d** Expression of LVBU and BCL6 were determined by qRT-PCR after RKO cells were transfected with miR-10a or miR-34c mimics and control microRNAs (*N* = 3 independent samples, *p < 0.05; **p < 0.01; ***p < 0.001). **e** Immunoblot analysis of BCL6 proteins after cells were transfected with the indicated siRNA and miRNA-inhibitors. **f** Immunoblot analysis of BCL6 proteins after cells were transfected with the indicated miRNA-mimics and LVBU overexpression plasmid. **g** Luciferase activities were detected by dual luciferase reporter assay after HEK-293T cells transfected with the indicated reporter plasmids (WT: inserted with miR-10a or miR-34c binding regions on LVBU sequence; MUT: inserted with mutated miR-10a or miR-34c binding regions on LVBU sequence) and microRNA mimics (*N* = 3 independent samples, *p < 0.05).

It is critical to characterize the mechanism behind LVBU-regulated BCL6 expression. Bioinformatic analysis showed that among those miRNAs binding with BCL6 or LVBU, miR-10, miR-30 and miR-34 are binding with both BCL6 and LVBU (Fig. S6a). Since LVBU and BCL6 were both abundant and upregulated in CRC, and BCL6 had been reported that it could be regulated by miR-10a, and miR-34c, we hypothesized that LVBU might regulate BCL6 mRNA expression through a miRNA-dependent mechanism. To test this hypothesis, we performed bioinformatic analysis and secondary structure prediction (Fig. S6b), and found that both miR-10a and miR-34c indeed have several binding sites within LVBU RNA and BCL6 3’UTR RNA sequence (Fig. 5a). RNA immunoprecipitation (RIP) results showed an enrichment of LVBU and BCL6 with AGO2 antibody, which indicated that LVBU and BCL6 were recruited to AGO2 related RNA-induced silencing complexes and might interact with miRNAs (Fig. 5b). Additionally, to test the direct interaction between miR-10a or miR-34c with LVBU and BCL6, biotin-labeled miRNA pull-down assays were performed to capture the putative binding RNAs using streptavidin-coated beads from cells transfected with 3’end-biotinylated miR-10a, miR-34c, or control miRNA. The results showed that both LVBU and BCL6 were significantly enriched in miR-10a or miR-34c captured fractions (Fig. 5c), suggesting that miR-10a and miR-34c are involved in regulating the link between LVBU and BCL6.

Furthermore, transient overexpression of miR-10a and miR-34c mimics led to reduction of both BCL6 and LVBU mRNA levels (Fig. 5d). Western blot results demonstrated that transient transfection of miR-10a or miR-34c inhibitors could reverse the downregulation of BCL6 caused by knockdown of LVBU (Fig. 5e). On the other hand, LVBU-mediated upregulation of BCL6 can be attenuated by miR-10a or miR-34c mimics (Fig. 5f). Finally, we found that reporter containing wild type miR-10a or miR-34c binding sites of LVBU showed a markedly lower luciferase activity in HEK-293T cells with either overexpression of miR-10a or miR-34c mimics, but mutational binding sites did not have this phenomenon (Fig. 5g). Taken together, these results demonstrated that LVBU positively regulates BCL6 expression through sponging miR-10a and miR-34c.

### Knockdown of LVBU mitigates tumor growth in vivo

**Fig. 6:**
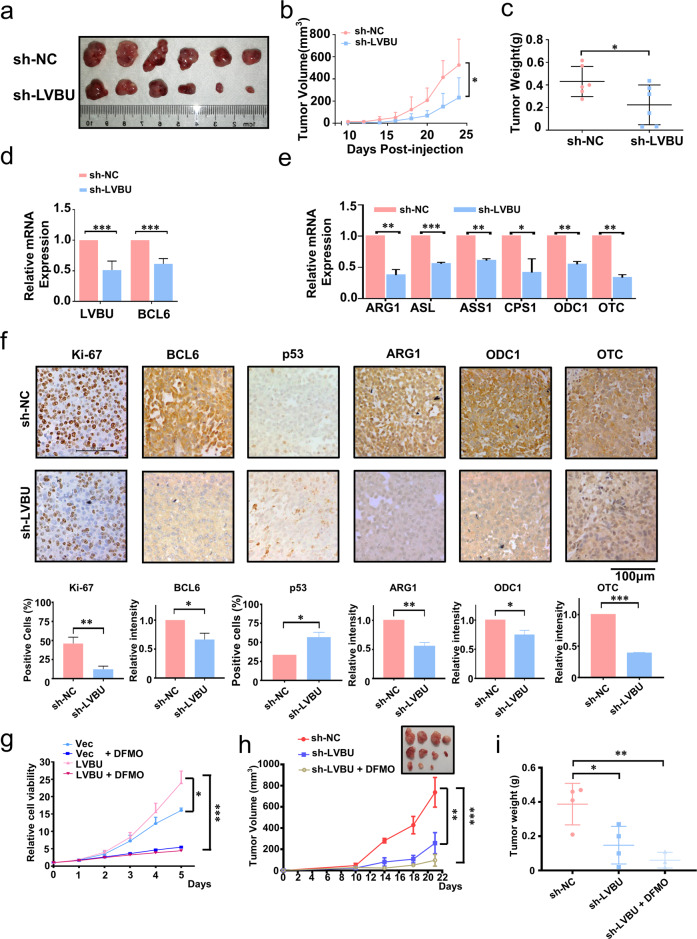
LVBU promotes tumorigenesis in vivo. **a**–**c** Dox-inducible HCT116-shLVBU cells were subcutaneously injected into nude mice. Mice was *i.p*. injected with PBS or Dox (to induce LVBU knockdown). Tumor growth curves and final tumor weight are shown (*N* = 6 independent tumors, *p < 0.05). **d** The mRNA expression of LVBU and BCL6 were detected by qRT-PCR in tumor tissues obtained from (**a**). (*N* = 6 independent samples, ***p < 0.001). **e** The mRNA expression of indicated urea cycle genes was detected by qRT-PCR in sh-NC or sh-LVBU tumor tissues obtained from (**a**). (sh-NC, negative control. *N* = 3 independent samples, *p < 0.05; **p < 0.01; ***p < 0.001). **f** Representative IHC staining of Ki-67, BCL6, p53, ARG1, ODC1 and OTC in tumor tissues obtained from (**a**). The staining intensity of indicated proteins in (**f**) was quantitated by Image J and presented as bar graphs (*N* = 3 independent tumors, *p < 0.05; **p < 0.01; ***p < 0.001). **g** Relative cell proliferation rate was examined after cells were transfected with LVBU overexpression plasmids with or without DFMO treatment (*N* = 3 independent samples, *p < 0.05; ***p < 0.001). **h**, **i** Dox-inducible HCT116-shLVBU cells were subcutaneously injected into nude mice. Mice were i.p. injected with PBS or Dox or DOX + DFMO. Tumor growth curves and final tumor weight are shown (*N* = 4 independent tumors, *p < 0.05; **p < 0.01).

To determine the in vivo role of LVBU function in tumorigenesis, doxycycline (DOX) inducible LVBU stable knock down cells were subcutaneously implanted into nude mice to establish CRC mouse xenograft model. Mice bearing LVBU knockdown cells had reduced tumor growth when compared with sh-NC control (Fig. 6a–c). Quantitative RT-PCR analysis of mouse xenografted tumor tissues confirmed the knockdown efficiency of LVBU, with concurrent downregulation of BCL6 (Fig. 6d). Notably, LVBU Knockdown tumors also showed a decreased gene expression in urea cycle and polyamine related enzymes, including ARG1, ASL, ASS1, CPS1, ODC1, and OTC, based on quantitative RT-PCR assay (Fig. 6e). Consistently, immunohistochemistry staining results showed a pronounced decrease in the BCL6, Ki-67, and urea cycle related gene (ARG1, ODC1 and OTC) products in the tumor mass while p53 is upregulated in LVBU knockdown tumors (Fig. 6f).

Eflornithine (Difluoromethylornithine, DFMO) is a suicide inhibitor of ODC1. It is currently used in phase II clinical trial for relapsed/refractory neuroblastoma (NCT04301843) and in phase III clinical trial for preventing recurrence of high-risk adenomas and second primary colorectal cancers (NCT01349881) [24]. Significantly, we found that DFMO could abolish LVBU-mediated cell proliferation (Fig. 6g), suggesting that DFMO is efficient in targeting ODC1 activity regulated by LVBU expression. CRC xenograft mouse model showed that combined DFMO treatment and LVBU knockdown could further inhibit the tumorigenesis and tumor weight significantly (Fig. 6h, i).

### Blocking polyamine synthesis inhibits LVBU-high expression CRC malignant growth

**Fig. 7:**
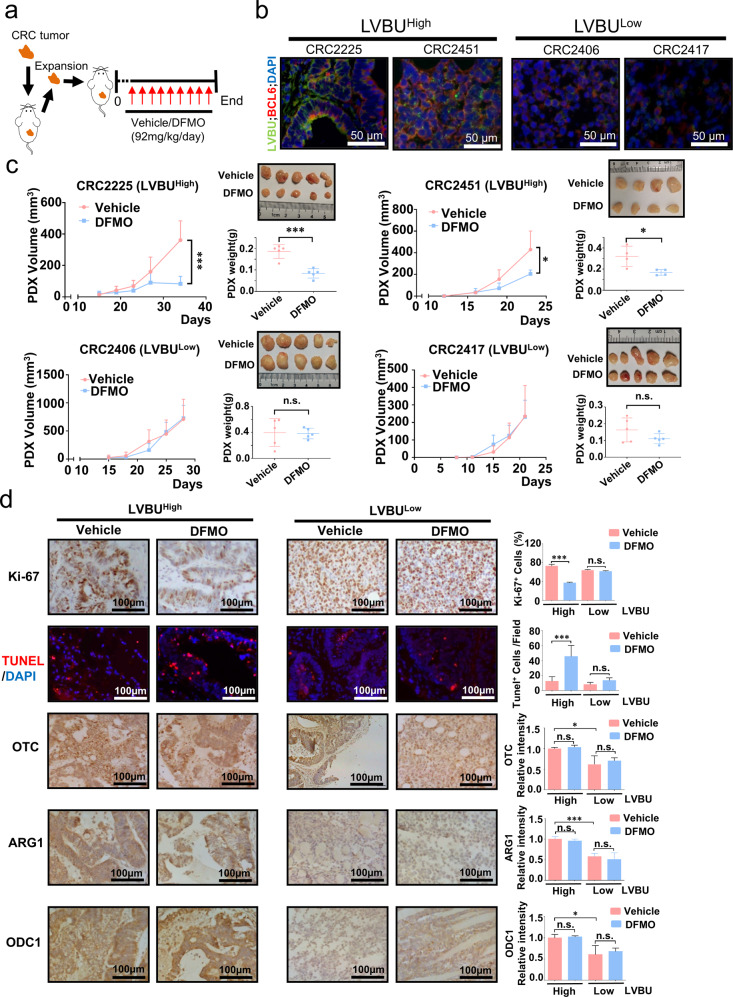
Impeding the urea cycle/polyamine synthesis suppresses LVBU-high CRC growth in PDXs. **a** Treatment schedule of ODC1 inhibitor DFMO is indicated. DFMO (Vehicle) was *i.p*. injected every day until the end of the experiments. **b** LVBU and BCL6 RNA fluorescence in situ hybridization (FISH) staining in indicated PDX tumor sections. **c** Tumor growth curve of immunocompromised mice engrafted with indicated CRC PDXs that express high or low LVBU. Representative xenograft images and statistical analysis of tumor volume and weight with or without DFMO treatment are shown (*N* = 4 or 5 independent tumors, *p < 0.05; ***p < 0.001). **d** IHC staining for Ki-67, OTC, ARG1, ODC1 and fluorescence TUNEL staining on indicated CRC PDX tumor sections. The staining intensity was quantitated by Image J and presented as bar graphs (*N* = 3 independent experiments, *p < 0.05; ***p < 0.001).

To harness the functions of LVBU-mediated urea cycle /polyamine synthesis in tumor formation for exploring strategies in potential clinical cancer therapy, we established patient-derived xenografts (PDX) model (Fig. 7a) [25]. Fresh primary tumor samples resected from CRC patients (LVBU high or LVBU low) were implanted into the immunocompromised mice. LVBU high CRC samples indeed contain high BCL6 levels, while LVBU low CRC samples have reduced BCL6 expression based on RNA fluorescence in situ hybridization (FISH) staining (Fig. 7b) and quantitative RT-PCR assay (Fig. S6c). We employed DFMO for the PDX treatment efficacy assay. The mice were *I.P*. injected with either vehicle or DFMO when the PDX tumors were palpable. Significantly, administration of DFMO in the established LVBU-high PDX xenografts suppressed tumor growth efficiently (Fig. 7c). By contrast, DFMO had no significant impact on the growth of LVBU-low PDX xenografts (Fig. 7c). Immunohistochemistry Ki-67 and fluorescence TUNEL staining showed that DFMO treatment could significantly inhibit tumor cells growth and induce more apoptosis in LVBU-high PDX xenografts but not in the LVBU-low PDX xenografts (Fig. 7d). While OTC, ARG1 and ODC1 levels were higher in LVBU-high PDX xenografts than those of LVBU-low PDX as expected, expression levels of these proteins are not affected by DFMO treatment (Fig. 7d). Obviously, DFMO is very specific in inhibiting ODC1 activity without changing ODC1 protein expression level in LVBU-high CRC.

Together, our animal model data demonstrates that the pivotal role of LVBU in activating UC/polyamine synthesis pathway and in promoting tumor cell growth can be recapitulated in vivo. Our PDX model experiments also point out that inhibiting polyamine biosynthetic pathway is effective in suppressing tumors with high expression level of LVBU.

## Discussion

**Fig. 8:**
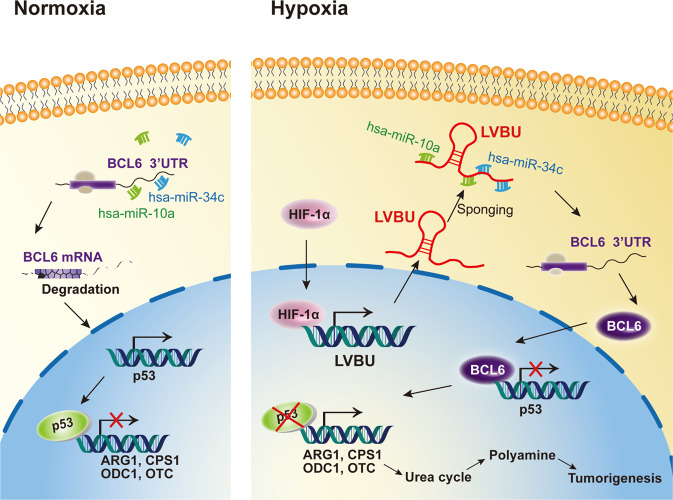
HIF-1α-induced LVBU promotes CRC tumorigenesis through sponging miR-10a/34c and impacts BCL6-p53 axis to regulate urea cycle/polyamine synthesis. Upon the induction of LVBU by hypoxia, BCL6 is no longer vulnerable to miR-10a/34c-mediated degradation due to LVBU’s sponging effect and is therefore stabilized, which in turn attenuates the transcriptional repressing activity of p53 to allow the expression of *ARG1*, *CPS1*, *OTC*, and *ODC1*, thereby promoting tumorigenesis via regulating UC/polyamine synthesis.

Cancer metabolic reprogramming is characterized as a pivotal hallmark of cancers as the high energetic/anabolic needs of tumor cell growth. Deregulations of oncogenes or tumor suppressor genes lead to high cellular bioenergetics that allows a growth advantage by supplying fuel, building block, or changing gene expression level for cancer cell growth [26]. Under these important needs, ammonia is largely generated during increased metabolic processing. Importantly, the ammonia needs to be eliminated to keep the condition for continuing tumor growth. Our studies indicate that LVBU is induced by hypoxia and is upregulated in colorectal cancer. In searching for a potential impact of LVBU-overexpression in CRC, we found that deregulation of urea cycle to be the culprit. This reprogramming is abnormally regulated through preserving BCL6-mediated p53 transcriptional repression and subsequent suppressing p53-mediated repression of transcripts of urea cycle enzymes (CPS1, OTC and ARG1) and polyamine biosynthesis enzyme (ODC1) (Fig. 8). Therefore, LVBU positively regulates urea cycle to dispose ammonia and is involved in urea cycle/polyamine biosynthesis.

HIF-1α is a critical transcription factor in mediating cancer metabolic reprogramming in response to hypoxia, including glycolysis and oxidative phosphorylation [12, 27, 28]. However, its role in urea cycle and polyamine biosynthesis has not been elucidated. We revealed that HIF-1α induced the expression of LVBU through promoter binding/activation, which positively enhanced the urea cycle through p53 pathways. Our findings demonstrate for the first time that HIF-1α-LVBU-p53 axis in mediating urea cycle for promoting cancer growth. On the basis of its important functions in cancers, HIF-1α is an important therapeutic target for cancer treatment [29]. However, no HIF-1α inhibitor has been clinically approved so far [29]. Giving its effect in regulating LVBU and urea cycle in cancer, reemphasizing the need of developing HIF-1α inhibitors in cancer therapeutic design is necessary.

p53 regulates ammonia metabolism via downregulating transcription of CPS1, OTC and ARG1, thereby repressing the urea cycle [16]. p53′s role in suppressing ureagenesis and subsequent elimination of ammonia is critical for inhibition of tumor growth. Our data showed that LVBU increased BCL6 expression through sponging miR-10a/miR-34c. Our data also demonstrated that increased BCL6 can suppress p53 transcriptional activity, thereby possibly reducing p53-mediated expression of miR-34c, a target known to be induced by p53 [30]. Thus, a feedback loop between BCL6 and p53 is established. It is worthwhile to point out that LVBU’s capability in keeping miR-34c at bay is to strengthen the impact of BCL6 in reducing p53 activity. This is very significant for LVBU to manifest its oncogenic activity as miR-34 family is induced by p53 and functions as a tumor suppressor [30]. For example, lower level expression of miR-34 a/b/c is observed in ovarian cancers [31]. Also, miR-34 functions as a tumor-suppressor and prognostic biomarker in gastric cancer [30]. BCL6 binds the p53 promoter and suppresses p53 expression. Germinal-centre B cells have high levels of BCL6, thus p53 expression is compromised. Importantly, BCL6 is implicated in the pathogenesis of human lymphomas, such as the diffuse large B-cell type [32]. It is then conceivable that LVBU’s positive impact on BCL6 will lead to tumorigenesis such as the case in lymphoma. More potential cancer studies are warranted.

miR-10a′s negative impact on BCL6 suggests that it will have a role in cancer. It has been shown that miR-10a targets BCL6 transcript in the 3′-untranslated region (3′-UTR), thereby attenuating the expression of BCL6 [33]. The relationship between miR-10a and BCL6 is consistent with our observation that LVBU sponges miR-10a to preserve the expression of BCL6. Other miRNAs can also regulate BCL6 such as miR-144-3p, which is down-regulated in CRC [34]. miR-144-3p can suppress cell proliferation and cause G1/S cell cycle arrest. It remains to be determined whether this miRNA is participating in LVBU’s effect in regulating BCL6 and urea cycle.

Due to the oncogenic driver role of BCL6 in cancer, many compounds were designed to interfere with its transcriptional repressor activity and subsequently attenuate the oncogenic effects of BCL6 [35]. Indeed, some BCL6-degrading compounds were discovered, and these compounds can induce expression of BCL6-repressed genes and triggers anti-proliferative effects [35]. For examples, BI-3802, a small molecule binding to the Broad-complex, Tramtrack and Bric-à-brac (BTB) domain of BCL6, can cause polymerization and the proteasomal degradation of BCL6 [36]. Further, FX1 interferes with the formation of the BCL6/repressor complex, reactivating the expression of BCL6-targeted genes [37]. Giving the LVBU-BCL6-p53 link, it is worthwhile to investigate these BCL6 inhibitors for treating LVBU-overexpressing cancers.

Polyamines, including putrescine, spermidine and spermine, are synthesized from ornithine through a series of decarboxylation and condensation processes. Polyamines are cationic chaperones that support *MYC* oncogenic activities through ionic and covalent mechanisms [38]. Thus, polyamine depletion can lead to anti-tumor activities, such as induction of protein translation stress or thymidine depletion stress. Expression studies in various tumors demonstrated that high expression of polyamine synthetic genes (such as *ODC1*, *AMD1*, *SRM*, *SMS*, and *AZIN1*) and low expression of polyamine catabolic genes (such as *SAT1*, *PAOX*, *OAZ1*, *OAZ2*, and *OAZ3*) correlate with poor clinical outcome [39].

Our studies indicated that LVBU increased ODC1 expression via downregulating p53 to promote polyamine biosynthesis and cancer growth. Interestingly, ODC1 is a gene target of MYC. *ODC1* gene is co-amplified with *MYCN* gene in about 15–20% of *MYCN*-amplified tumors [38]. Further, high ODC1 expression is correlated with poor clinical outcome in cancers, suggesting that it can serve as a drug target for cancer therapy. Of note, p53 can downregulate Myc expression at posttranscriptional level through activating 14-3-3σ-FBW7 axis to mediate Myc ubiquitination and degradation [40]. In addition to LVBU-p53’s direct impact on ODC1 transcriptionally, it is conceivable that LVBU–p53 axis can also downregulate MYC protein, thereby subsequently suppressing ODC1 gene expression.

Because of the importance of ODC1 in polyamine synthesis, drugs were designed to inhibit its activity for disease treatments. For example, DFMO, an FDA-approved drug, is an irreversible inhibitor of ODC1 and can inhibit ODC1 activity [20, 39]. DFMO was approved for the treatment of gambiense encephalitis (African sleeping sickness) caused by Trypanosoma brucei as polyamines are critical in the proliferation of these protozoa. Nonetheless mammalian cancer cells also need polyamines for proliferation. Thus, administrating ODC1 inhibitor such as DFMO in cancer can be a good treatment regimen. Our CRC xenograft model studies indicate that the role of LVBU in promoting cell proliferation, causing deregulation of p53-mediated urea cycle, and facilitating polyamine biosynthesis can be recapitulated in vivo, and that DFMO’s treatment efficacy in suppressing cancer can be explored further in CRC. Indeed, we further showed that cancer samples from LVBU-high CRC patients confer a vulnerability to DFMO treatment in PDX animal experiments, suggesting a potential clinical application for CRC patients with LVBU deregulation. Our studies underscore the pivotal LVBU-BCL6-p53-UC axis deregulation during tumor development and illustrate the potential of exploiting this axis to control urea cycle/polyamine biosynthesis deregulation for cancer treatment.

The colorectal cancer tissue sample size analyzed in this study is small. Also, whether LVBU can regulate urea cycle/polyamine biosynthesis in p53 null or mutated colorectal cancer cells is warranted to be investigated. In summary, our study provides insight into the complicated control of urea cycle and polyamine synthesis by identifying lncRNA LVBU as a critical regulator.

## Conclusions

This study demonstrated that LVBU, a novel HIF-1α-induced lncRNA, is upregulated and correlated with poor prognosis in CRC. LVBU could promote CRC cells proliferation and tumorigenesis in vitro and in vivo through regulating BCL6-p53 axis and subsequent urea cycle/polyamine synthesis pathway to promote CRC progression. LVBU may serve as a biomarker or therapeutic target of CRC.

## Material & methods

### Patients and specimens

Thirty-two CRC tumor tissues and matched non-tumorous adjacent colorectal tissues were collected from the Department of Surgery at the Sixth Affiliated Hospital of Sun Yat-sen University. A CRC cDNA microarray (# HColA095Su02) was purchased from SHANGHAI OUTDO BIOTECH CO., LTD (Shanghai, China), which contains 80 CRC cases and the related clinical and survival information. Only colorectal adenocarcinoma cases were included in the survival analysis. And one case with undetectable LVBU expression was excluded from the survival analysis. All experiments were approved by the Ethics Committee of The Sixth Affiliated Hospital of Sun Yat-sen University.

### Chromatin immunoprecipitation (ChIP) assays

CHIP assays were performed as previously described [41]. Briefly, RKO cells were harvested for CHIP assay after cultured in hypoxia condition. IgG (5 µg per reaction; Millipore, #12-370) and HIF-1α antibody (5 µg per reaction; Genetex, #GTX127309) were used in the assay. And the CHIP assay was performed as the manual described (Millipore, # 17-10085). The primers specific for HIF-1α promoter are shown in Supplementary Table S4.

### LVBU and BCL6 RNA fluorescence in situ hybridization (FISH) staining

RNA FISH staining was performed on CRC patients’ derived xenograft (PDX) tissues using RNA Fluorescence In Situ Hybridization Kit as the manual described (Exon Biotechnology Inc, Guangzhou, China) [42]. LVBU probe (labeled with digoxin) is designed to target the 2563-3034 bp region of ENST00000647108.1. BCL6 probe (labeled with biotin) is designed to target the 1378-1957bp region of ENST00000406870.7. After incubation with anti-digoxin and avidin-HRP secondary antibodies, tyramide signal amplification (TSA) was used to develop the fluorescence signal and the nucleus was stained by DAPI. Finally, the images were captured by microscope (Nikon 80i, Japan).

### RNA pull down

Biotin labeled miR-10a, miR-34c and negative control mimics were purchased from Shanghai Genepharma company. Briefly, 5 × 10^6^ HCT116 cells were transfected with 600 pmol indicated biotin labeled miRNAs by using Lipofectamine 2000. Twenty-four hours later, after washing twice with PBS, the cells were harvested with lysis buffer (5 mM MgCl_2_, 100 mM KCl, 20 mM Tris (pH7.5), 0.3% NP-40, 50U of RNase OUT (Invitrogen, USA), complete protease inhibitor cocktail (Roche Applied Science, IN)) on ice for 10 min. After centrifugation at 12,000 g for 10 min, the supernatant was used for miRNA biotin pull down experiments according to previous reports [43]. The mRNA level of LVBU and BCL6 after miRNA pull down were quantified by real-time PCR.

### Statistical analysis

Results are presented as the means±S.D (at least three biological replicates or three independent experiments). Sample size calculation was performed for evaluating LVBU expression in CRC patients’ tumor tissues (α = 0.05, β = 0.1). Differences between two groups were analyzed using Student’s *t*-test or paired *t*-test (two-tailed, with *p* ≤ 0.05 considered significant). One-way analysis of variance followed by Bonferroni test was performed for comparisons among multiple groups. Variances were similar between all the statistically compared groups. Cumulative survival was evaluated using the Kaplan-Meier method (log-rank test). For animal studies, a priori sample size calculations were not performed. All the statistical analyses were performed using the SPSS 16.0 and PASS software.

## Supplementary information

Supplementary materials and methods

Supplemental tables s1-s4

Supplementary figure legends

Supplementary Figure 1

Supplementary Figure 2

Supplementary Figure 3

Supplementary Figure 4

Supplementary Figure 5

Supplementary Figure 6
