# Supplementary material for: Roles of lncRNA LVBU in regulating urea cycle/polyamine synthesis axis to promote colorectal carcinoma progression
Source: Oncogene. 2022 Jul 29;41(36):4231–43. doi: 10.1038/s41388-022-02413-8 (PMC9439952; doi:10.1038/s41388-022-02413-8)
Supplement: Supplementary file 1 — Supplementary materials and methods [file 41388_2022_2413_MOESM1_ESM.docx]

**Supplemental Material & Methods**

***Cell culture, reagents and transfection***

All the cells were obtained from ATCC, and maintained at 37℃ and 5% CO_2_ (Thermo, Waltham, MA, USA). For hypoxia treatment, the cells were cultured in 1% O_2_ conditions. HEK-293T was cultured in Dulbecco’s modified Eagle’s medium (DMEM) with 10% fetal bovine serum. HCT116 cells were cultured in RPMI-1640 medium with 10% fetal bovine serum. RKO cells were cultured in modified Eagle’s medium (MEM) with 10% fetal bovine serum. Small interfering (si) RNA duplexes and antisense oligonucleotide (ASO) were designed and synthesized by Genepharma (Shanghai, CN). miR-10a and miR-34c mimics, inhibitors and corresponding negative control (NC) were synthesized by Ribobio (Guangzhou, CN). The sequences are shown in Supplementary Table S2. All transient transfections of plasmids, siRNA, ASO, microRNA mimics and inhibitors were followed the standard protocol for Lipofectamine 2000 transfection reagent (Invitrogen, CA, USA)

***Plasmids construction***

pGL3-basic, pGL3-control, pGL4.73 and PG13-luc (p53 reporter plasmid) were kept in our lab. LVBU and BCL6 were amplified from 293T cells by PrimeSTAR® MAX DNA polymerase (Takara, Kyoto, JPN). LVBU was subcloned into the AgeI and EcoRI sites of the pEYFP-C1-tag vector (Citrine sequence was replaced), while BCL6 was subcloned into the BamHI and HindIII sites of pcDNA3.1-myc vector. Primers for wild type and mutation cloning of LVBU promoter (pGL3-LVBU promoter BR-1, pGL3-LVBU promoter BR-2, pGL3-LVBU promoter BR-3, pGL3-LVBU promoter MUT A, pGL3-LVBU promoter MUT B, pGL3-LVBU promoter MUT C, pGL3-LVBU promoter MUT D, pGL3-LVBU promoter MUT E, pGL3-LVBU promoter MUT ALL), wild type binding sites of miR-10a and miR-34c are shown in Supplementary Table S3. shRNA sequences of Dox inducible knockdown plasmids (HIF-1α, LVBU, BCL6) are shown in Supplementary Table S2. All enzymes were purchased from New England Biolab (MA, US).

***RNA-seq analysis***

Total RNA was extracted using Trizol reagent (Invitrogen, CA, USA) and then sent to the company (Majorbio, Shanghai, CN) for mRNA-seq analysis, and the company (Biomedlab, Shanghai, CN) for whole transcriptome sequencing.

***5’ and 3’ rapid amplification of cDNA Ends (RACE)***

5’RACE and 3’RACE assays were carried out using the SMARTer™ RACE cDNA kit (Clontech) according to the manufacturer’s instructions. The sequences of the gene specific primers (GSP) used for PCR are listed in Supplementary Table S3.

***Real-time quantitative PCR (qPCR)***

Total RNA was extracted from cells using Trizol reagent (Invitrogen, CA, USA) and then reverse transcribed with ReverTra Ace® qPCR RT Master Mix with gDNA Remover (TOYOBO, Osaka, JPN). Real-time quantitative PCR was carried out using 2× SYBR Green qPCR Master Mix (Bimake, Shanghai, CN) in a LightCycler® 480 II instrument (Roche, Basel, Switzerland). A list of primers used for real-time quantitative PCR experiments are shown in Supplementary Table S4.

***Western blot***

Total cellular proteins were extracted and separated via sodium dodecyl sulfate polyacrylamide gel electrophoresis. And the proteins were transferred to polyvinylidene difluoride membranes (Millipore, Mass, USA). The membranes were blocked with 5% nonfat milk for 1 hour at room temperature prior to incubation with indicated primary antibodies. Subsequently membranes were washed and incubated for 1 hour at room temperature with peroxidase-conjugated secondary antibodies (Thermo Scientific). Following several washes, chemiluminescent images of immunodetected bands on the membranes were recorded on X-ray films using the enhanced chemiluminescence (ECL) system (Millipore). Antibodies against BCL6 (Boster, #BM4070), p53 (Cell Signaling Technology, #2527S), p21 (Cell Signaling Technology, #2947S), ARG1 (Boster, #BM3973), ODC1 (Boster, #BM1322), OTC (Proteintech, #67553-1), HIF-1α (GeneTex, # GTX127309) and GAPDH (Proteintech, # 60004-1-Ig) were used in this study.

***Cell proliferation and colony formation***

For cell proliferation assays, a total of 3,000 cells were seeded into 96-well plates. Cell viability was quantified using the Cell Counting Kit-8 (APExBIO, Houston, USA) according to the manufacturer’s instructions. For colony formation assays, 500 cells were seeded in the 6-well plates and incubated with normal medium for 10 days. Clones were fixed and stained with 0.5% crystal violet, and the number of colonies was counted.

***Cell senescence assay***

For cell senescence assay, RKO cells treated with either si-NC or si-LVBU were seeded in 6-well plates under FBS-free cell culture medium. After 24 hours, cells were harvested for β-Galactosidase staining (Beyotime Biotechnology, Shanghai, CN) according to the manufacturer’s instructions.

***Cell cycle***

For cell cycle assay, RKO and HCT116 cells treated with either si-NC or si-LVBU were seeded in 6-well plates for 48h. After 48h, cells were collected for cell cycle analysis with Flow cytometry instrument.

***LC-MS***

Stable transfected CRC cells were cultured in 6-well plates with or without Dox for 48 hours. After 48 hours, cells were washed twice with ice cold PBS and collected with 200μL 80% prechilled HPLC-grade methanol. The samples were kept in -80ºC overnight. After centrifuge at 13,000r for 10 mins, the supernatant was transferred to a fresh glass vial for further LC-MS analysis.

The LC-MS analysis was performed by Sun Yat-sen University Instrumental Analysis & Research Center. Briefly, identification and quantification of the analyses were carried out on a UPLC-Q-TOF (Ultimate 3000, Thermo/timsTOF, Bruker Dalton). ACQUITY UPLC® BEH HILIC column，2.1 mm x 100 mm，1.7 μm (Waters) was used for LC separation, by using gradient elution with 0.1% formic acid acetonitrile as solvent A and 0.1% formic acid water as solvent B. The gradient program is as follows: 0-1 min 85% A, 1-5 min 85% A to 50% A, 5-7 min 50% A ,7-7.1 min 50% A to 85% A, 7.1-10 min 85% A. The flow rate was set at 0.4 mL min-1, and the injection volume was 5 μl. The total run time was 10 min for each sample. Detection was in positive ion mode using ESI ion source and the capillary voltage was 4500 V, dry temperature was held at 220 ºC, dry gas was 8.0 L min-1, and Nebulizer was 1.5 bar. The data were analyzed with the software MetaboScape 4.4 (Bruker).

***RNA stability assay***

Cells were treated with either si-NC or si-LVBU and seeded in 6-well plates for 48h. Before collecting cells, they were treated with 100ng/mL Actinomycin D (Sigma, CA, USA) for indicated time (2h, 4h, 6h, 8h, and 10h). After 48h, total RNA was extracted using Trizol reagent (Invitrogen, CA, USA) and RT-qPCR detect the remaining p53 mRNA. p53 mRNA stability in si-NC group and si-LVBU group were compared by GraphPad Prism 7.00.

***RNA immunoprecipitation***

RNA immunoprecipitation (RIP) experiments were performed with a Magna RIP™ RNA-Binding Protein Immunoprecipitation Kit ((Millipore, #17-700) according to the manufacturer’s instructions. Briefly, HCT116 or RKO cells were harvested by RIP lysis buffer. And the interested RNA and protein complex were immunoprecipitated with AGO2 antibody (Cell Sinaling technology, #2897S) and protein A/G magnetic beads. After washing off unbound materials, bingding RNA was extract with the phenol:chloroform:isoamyl alcohol (125:24:1, pH = 4.3). Co-precipitated RNAs were detected by real-time PCR. IgG was used as negtive control and the gene-specific primers used for detecting LVBU and BCL6 were presented in supplementary table S3.

***Luciferase reporter assay.***

Constructed luciferase reporter plasmids and pGL4.73(control renilla plasmids) were transfected into HEK293T cells after the indicated treatment. Forty-eight hours later, the luciferase activity was measured using the Dual-Luciferase Reporter Assay System (Promega, Madison, WI, USA), and normalized to renilla luciferase activity[[1](#_ENREF_1)].

***Animal studies***

The animal studies were approved by the Animal Ethical and Welfare Committee of the Sixth Affiliated Hospital of Sun Yat-sen University. All the experiments were performed in a completely blinded manner. Mouse xenograft cancer studies were performed as study described before[[2](#_ENREF_2), [3](#_ENREF_3)]. Female athymic BALB/c nude mice (5 weeks old) were used for animal studies. RKO cells (1×10^7^) were injected subcutaneously into the hind-flanks of mice to establish the CRC xenograft model. Mice were randomly divided into two groups. Either PBS or doxycycline (Sigma, CA, USA)) was administered by intraperitoneal injection. Tumor length and width were measured twice weekly, and the volume was calculated according to the formula (length × width^2^)/2. At the end of the experiments, the xenografts were collected for further analysis.

For the patient-derived xenograft (PDX) study, PDX models were established at the Sixth Affiliated Hospital of Sun Yat-sen University and were performed as previously described before [[4](#_ENREF_4)]. Patient-derived tumor fragments (2-3 mm^3^) were surgically embedded under the skin of NCG mice. Mice were assigned randomly into two treatment groups when tumors reached approximately 50 mm^3^.

***Immunohistochemistry (IHC)***

Paraffin-embedded sections were subjected to immunohistochemistry staining[[5](#_ENREF_5)]. Antibodies against BCL6 (Boster, #BM4070), p53 (Cell Signaling Technology, #2527S), ARG1 (Boster, #BM3973), ODC1 (Boster, #BM1322), OTC (Proteintech, #67553-1) and Ki-67 (Cell Signaling Technology, #9449) were used respectively. Immunohistochemistry was performed using a standard protocol with a commercial kit (DAKO, Carpinteria, CA).

**References:**

1 Yu H, Xie M, Meng Z, Lo CY, Chan FL, Jiang L *et al*. Endolysosomal ion channel MCOLN2 (Mucolipin-2) promotes prostate cancer progression via IL-1beta/NF-kappaB pathway. *British journal of cancer* 2021; 125: 1420-1431.

2 Geng A, Tang H, Huang J, Qian Z, Qin N, Yao Y *et al*. The deacetylase SIRT6 promotes the repair of UV-induced DNA damage by targeting DDB2. *Nucleic acids research* 2020.

3 Velazquez-Torres G, Fuentes-Mattei E, Choi HH, Yeung SJ, Meng X, Lee MH. Diabetes mellitus type 2 drives metabolic reprogramming to promote pancreatic cancer growth. *Gastroenterology report* 2020; 8: 261-276.

4 Li K, Wu JL, Qin B, Fan Z, Tang Q, Lu W *et al*. ILF3 is a substrate of SPOP for regulating serine biosynthesis in colorectal cancer. *Cell Res* 2020; 30: 163-178.

5 Meng X, Vander Ark A, Lee P, Hostetter G, Bhowmick NA, Matrisian LM *et al*. Myeloid-specific TGF-beta signaling in bone promotes basic-FGF and breast cancer bone metastasis. *Oncogene* 2016; 35: 2370-2378.
