# Supplementary material for: Roles of lncRNA LVBU in regulating urea cycle/polyamine synthesis axis to promote colorectal carcinoma progression
Source: Oncogene. 2022 Jul 29;41(36):4231–43. doi: 10.1038/s41388-022-02413-8 (PMC9439952; doi:10.1038/s41388-022-02413-8)
Supplement: Supplementary file 2 — Supplemental tables s1-s4 [file 41388_2022_2413_MOESM2_ESM.docx]

Supplemental Tables

**Table S1. Correlation of LVBU expression in tissues with CRC patient clinicopathological variables**

|  |  | **LVBU** | |  |
| --- | --- | --- | --- | --- |
| **Variables** | **Case #^κ^** | **Low expression** | **High expression** | ***P v*alues^§^** |
| Age (yr) |  |  |  |  |
| <59 | 55 | 28 (50.9%) | 27 (49.1%) | 0.99 |
| ≥59 | 24 | 12 (50.0%) | 12 (50.0%) |  |
| Gender |  |  |  |  |
| Male | 46 | 20 (43.5%) | 26 (56.5%) | 0.17 |
| Female | 33 | 20 (60.6%) | 13 (39.4%) |  |
| Histologic grade |  |  |  |  |
| Well/moderate | 60 | 28 (46.7%) | 32 (53.3%) | 0.42 |
| Poor | 18 | 11 (61.1%) | 7 (38.9%) |  |
| pT category |  |  |  |  |
| T1+T2 | 4 | 2 (50.0%) | 2 (50.0%) | 0.99 |
| T3+T4 | 70 | 35 (50.0%) | 35 (50.0%) |  |
| pN status |  |  |  |  |
| N0 | 40 | 19 (47.5%) | 21 (52.5%) | 0.65 |
| Nx （≥1） | 35 | 19 (54.3%) | 16 (45.7%) |  |
| Distant metastasis |  |  |  |  |
| M0 | 78 | 40 (50.6%) | 39 (49.4%) | 0.49 |
| M1 | 1 | 0 ( 0.0%) | 1(100.0%) |  |
| Clinical stage |  |  |  |  |
| I + II | 40 | 18 (45.0%) | 22 (55.0%) | 0.65 |
| III + IV | 35 | 19 (54.3%) | 16 (45.7%) |  |
| Tumor type |  |  |  |  |
| Adenocarcinoma | 73 | 36 (49.3%) | 37 (50.7%) | 0.99 |
| Mucinous | 6 | 3 (50.0%) | 3 (50.0%) |  |

**§** χ^2^ test

**κ** Exclude cases lack of information

**Table S2. Oligonucleotide sequences**

| **Name** | **Sequence** |
| --- | --- |
| ASO-LVBU | UCCCAGGUAAAGACGAACGG |
| si-LVBU | GCAGUACUGUGCUCACCUATT |
| miR-10a mimics | CAAAUUCGUAUCUAGGGGAAUA |
| miR-34c mimics | AAUCACUAACCACACGGCCAGG |
| mimics control | UCACAACCUCCUAGAAAGAGUAGA |
| miR-10a inhibitors | UAUUCCCCUAGAUACGAAUUUG |
| miR-34c inhibitors | CCUGGCCGUGUGGUUAGUGAUU |
| inhibitor control | UCUACUCUUUCUAGGAGGUUGUGA |
| Dox inducible shRNA sequences |  |
| Sh-HIF-1α | **TOP:**CCGGCGGCGAAGTAAAGAATCTGAACTCGAGTTCAGATTCTTTACTTCGCCGTTTTT |
| Sh-BCL6 | **TOP:**CCGGGACACGGATCTGAGAATCTCTCGAGAGATTCTCAGATCCGTGTCTTTTTG |
| Sh-LVBU | **TOP:**CCGGGCAGTACTGTGCTCACCTACTCGAGTAGGTGAGCACAGTACTGCTTTTTG |

**Table S3. Primers sequence for cloning**

| **Name** | **Sequence** |
| --- | --- |
| LVBU full length | F: CCGCAGCGGCTAGAGGTTCCATTG |
|  | R: AACAAACAGAATCCAATCCACCAG |
| BCL6 full length | F: GCCTCGCCGGCTGACAGCTGTA |
|  | R: TCAGCAGGCTTTGGGGAGCT |
| LVBU promoter |  |
| BR-1 | F: GTGAAGGCAGTGAGTGGCCTG |
|  | R: TGGCTTCTTATTTTTAGCTGAATTAAA |
| BR-2 | F：CTGGCGCTCTCCTTTCCCTG |
|  | R: GTAGTCGAGGCTGCGTCACT |
| BR-3 | F：GTCCTGGAACCCCAGCGTTG |
|  | R: AGACTCTGCGCACGCGCA |
| MUT A | F:TTCATCTTAACCACCGTCTGCGGGAAGCCGCCCC |
|  | R: AGACTCTGCGCACGCGCA |
| MUT B | F:GCGG TTCATCTTAACCGGCGGCCGCGCCCCGCC |
|  | R: CCGGTTAAGATGAACCGCCCGGGGCCATGGCGA |
| MUT C | F：CGGAATTCATCTTCGCCCCCGTGAAATGCGTTC |
|  | R:GGGCGAAGATGAATTCCGGGGCGGGGCGCGGCC |
| MUT D | F：TCGCTTCATCTTAATGCGTTCCCAGTAGCCACG |
|  | R: GCATTAAGATGAAGCGGGGCGTGGTTCCGGGGC |
| MUT E | F：GTCCTGGAACCCCAGCGTTG |
|  | R: TTAAGATGAAGTTCCCCCTCCCGCCCCTGCT |
| MiRNA binding sites on LVBU |  |
| miR-10a binding sites | F: GGGAGTTTCCACCAGGGAGT |
|  | R: GGGACAGCTCCCTCGTCTCC |
| miR-34c binding sites | F: TGACACTCCCACGGACAGGC |
|  | R: AACCATTGACATCTGCAAGAAGTATC |
| RACE primers |  |
| 5’ GSP primer | CCAAGCGTTTGTCCATCATGTGGCATTTCTGGG |
| 3’GSP primer | GTCCAGACAGAGGCGGTATTGCCCG |

**Table S4. Primers for qRT-PCR**

| **Name** | **Sequence** |
| --- | --- |
| LVBU | F: CCGAAGCAGAAATCCATAGAA |
|  | R: ACCCTGAGTCCTCACAATAGA |
| U1 | F: GGAGATACCATGATCACGAAGG |
|  | R: CCACAAATTATGCAGTCGAGTTT |
| U6 | F: CTCGCTTCGGCAGCACA |
|  | R: AACGCTTCACGAATTTGCGT |
| β-Actin | F: CATGTACGTTGCTATCCAGGC |
|  | R: CTCCTTAATGTCACGCACGAT |
| BCL6 | F: CATGTACACATCTCGGCTCAA |
|  | R: CTCTGCTTCACTGGCCTTAATA |
| p53 | F: GGAAATTTGCGTGTGGAGTATTT |
|  | R: GTTGTAGTGGATGGTGGTACAG |
| p21 | F: GTCACTGTCTTGTACCCTTGTG |
|  | R: GGCGTTTGGAGTGGTAGAAA |
| CPS1 | F: CAACCTGGCAGTTCCTCTATAC |
|  | R: CTCATCCAAGACAGCTGAGAAG |
| OTC | F: ATGGGAACAATATCCTGCACTC |
|  | R: AGTCAGAGGCAGCAACTTTAG |
| ODC1 | F: GCTTTCACGCTTGCAGTTAATA |
|  | R: CACTCGACTCATCTTCGTCATC |
| ASS1 | F: CCCGCAAACAAGTGGAAATC |
|  | R: CCAGGGAGCAATGACCTTTAT |
| ASL | F: GTCATCTCTACGCTGCAGATTC |
|  | R: CCTTTGCGGACCAGGTAATAG |
| ARG1 | F: CCCTTTGCTGACATCCCTAAT |
|  | R: GGCTGATTCTTCCGTTCTTCT |
| PRDM1 | F: CAGGTCTGCCACAAGAGATTTA |
|  | R: CACCTTGCATTGGTATGGTTTC |
| RUVBL1 | F: GCTCTGGCTCTGGCTATTG |
|  | R: GCACCTCTGTCTTCTTGATCTC |
| YBX1 | F: CAGTATTCCAACCCTCCTGTG |
|  | R: CTGCGGAATCGTGGTCTATATC |
| CyclinE1 | F: CGGTATATGGCGACACAAGAA |
|  | R: GGTGCAACTTTGGAGGATAGA |
| CDK6 | F: TCACGAACAGACAGAGAAACC |
|  | R: CTCCAGGCTCTGGAACTTTATC |
| CDK2 | F: GCCTGATTACAAGCCAAGTTTC |
|  | R: AACAAGCTCCGTCCATCTTC |
| CyclinD1 | F: GTTCGTGGCCTCTAAGATGAAG |
|  | R: GATGGAGTTGTCGGTGTAGATG |
| CDK4 | F: ATGTGGAGTGTTGGCTGTATC |
|  | R: CAGCCCAATCAGGTCAAAGA |
| CHIP primers for HIF-1α | F: CAGCAGCAGGAAGACGAAG |
|  | R: GCCGTGGCTACTGGGAA |
