# Supplementary material for: Roles of lncRNA LVBU in regulating urea cycle/polyamine synthesis axis to promote colorectal carcinoma progression
Source: Oncogene. 2022 Jul 29;41(36):4231–43. doi: 10.1038/s41388-022-02413-8 (PMC9439952; doi:10.1038/s41388-022-02413-8)
Supplement: Supplementary file 3 — Supplementary figure legends [file 41388_2022_2413_MOESM3_ESM.docx]

**Figure S1. RACE analysis of the full length of LVBU.**

(a) Agarose gel electrophoresis of PCR products from 5’-RACE and 3’-RACE analysis of LVBU. (b) The nucleotide sequence of the full-length LVBU. (c) Schematic depicting identified LVBU genome sequence (including exons and introns) and the RACE primers sites**.**

**Figure S2. Detection of LVBU distribution in CRC cells.**

Distribution of LVBU in CRC cells. U1(nuclear) and β-actin (cytoplasm) were used as controls.

**Figure S3. LVBU promotes CRC cells proliferation.**

(a-c) Cell proliferation and foci formation, (d) Senescence (SA-β-gal staining) and (e) Cell cycle analysis (FACS) was performed in HCT116 and RKO cells after transfected with the indicated siRNA, antisense oligonucleotide (ASO) or expression plasmid (n=3 independent experiments, **p* < 0.05; ***p* < 0.01; ****p* < 0.001).

**Figure S4. LVBU regulates UC related genes expression.**

(a) Kaplan–Meier overall survival (OS) curves based on OTC expression in rectal carcinoma (READ) tissues from TCGA database. ROC curve was used to define the high and low expression group, and log-rank analysis was used to test for significance.

(b) Kaplan–Meier overall survival (OS) curves based on ASL expression in rectal carcinoma (READ) tissues from TCGA database. Tertile classification was used to define the high and low expression group, and log-rank analysis was used to test for significance.

(c) Knockdown of LVBU expression with ASO inhibited urea cycle related genes expression by qRT-PCR (n=3 independent experiments, **p* < 0.05; ***p* < 0.01).

**Figure S5. Hypoxia condition regulates UC related genes.**

(a) KEGG enrichment analysis of the top30 changed pathways after the cells under hypxoxia conditons at 12 and 24 hours.

(b) ARG1, ODC1 and OTC mRNA expression were detected by qRT-PCR in RKO and HCT116 cells after hypoxia treatment (n=3 independent experiments, **p* < 0.05; ***p* < 0.01; ****p* < 0.001).

(c) p53 mRNA expression was measured by qRT-PCR after the cells treated with DMSO or actinomycin-D (ActD). GAPDH was used as a loading control.

**Figure S6. Predicted miRNAs binding with BCL6 and LVBU.**

(a) Predited miRNAs binding with BCL6 and LVBU. TargetScan and DIANA web tools were used for the prediction.

(b) Predicted secondary structure of LVBU and miR-10a, miR-34c binding sites. RNAfold webServer was used for the prediction.

(c) LVBU mRNA expression was measured by qRT-PCR in CRC PDX tissue samples. LVBU are relatively high expressed in CRC2225 and CRC2451 PDX tissues and low in CRC2406 and CRC2417 PDX tissues (n=3 independent experiments, ****p* < 0.001).
