# Supplementary figures and images for: Roles of lncRNA LVBU in regulating urea cycle/polyamine synthesis axis to promote colorectal carcinoma progression
Source: Oncogene. 2022 Jul 29;41(36):4231–43. doi: 10.1038/s41388-022-02413-8 (PMC9439952; doi:10.1038/s41388-022-02413-8)

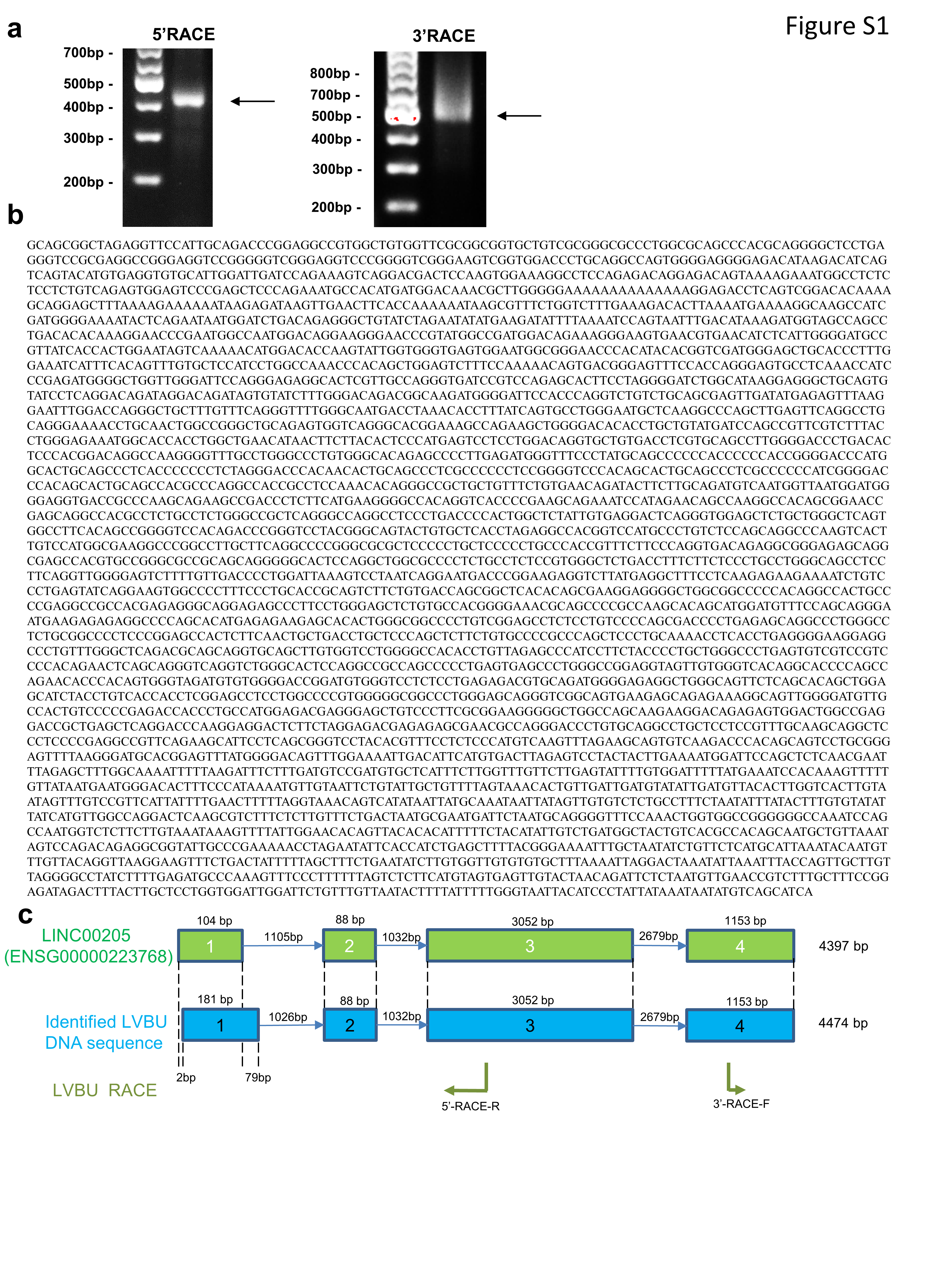

Supplement: Supplementary file 4 — Supplementary Figure 1 [file 41388_2022_2413_MOESM4_ESM.tif]

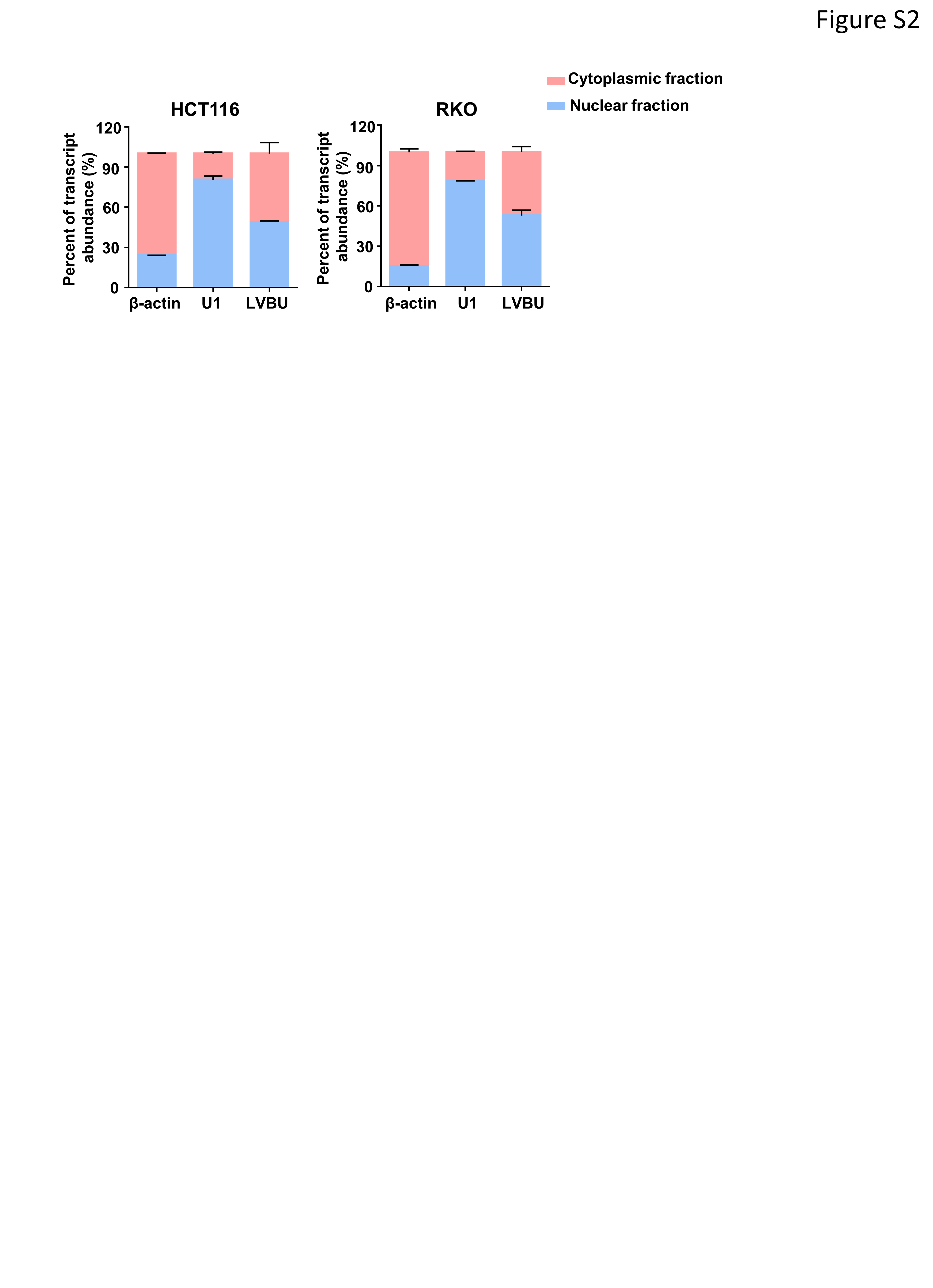

Supplement: Supplementary file 5 — Supplementary Figure 2 [file 41388_2022_2413_MOESM5_ESM.tif]

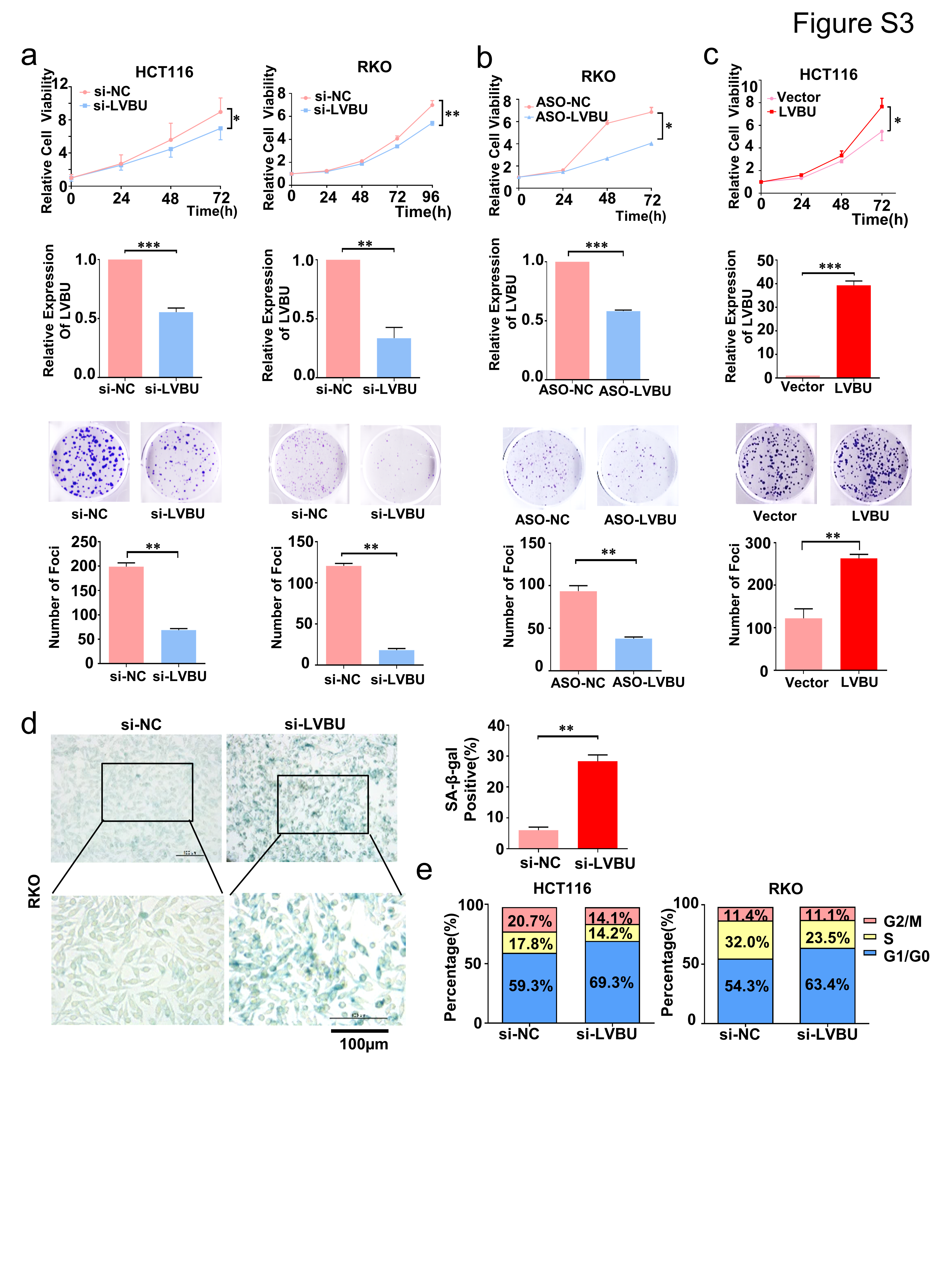

Supplement: Supplementary file 6 — Supplementary Figure 3 [file 41388_2022_2413_MOESM6_ESM.tif]

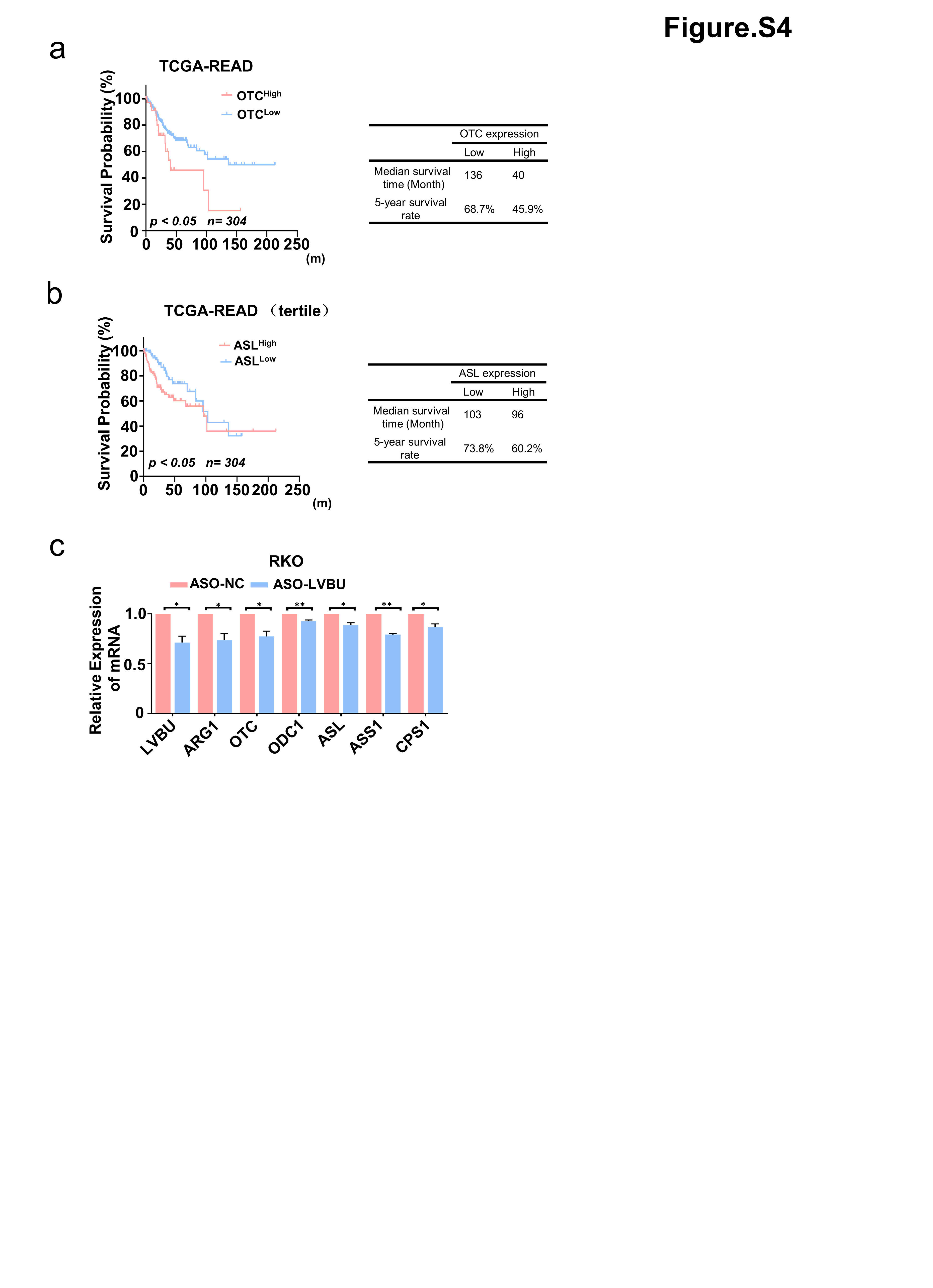

Supplement: Supplementary file 7 — Supplementary Figure 4 [file 41388_2022_2413_MOESM7_ESM.tif]

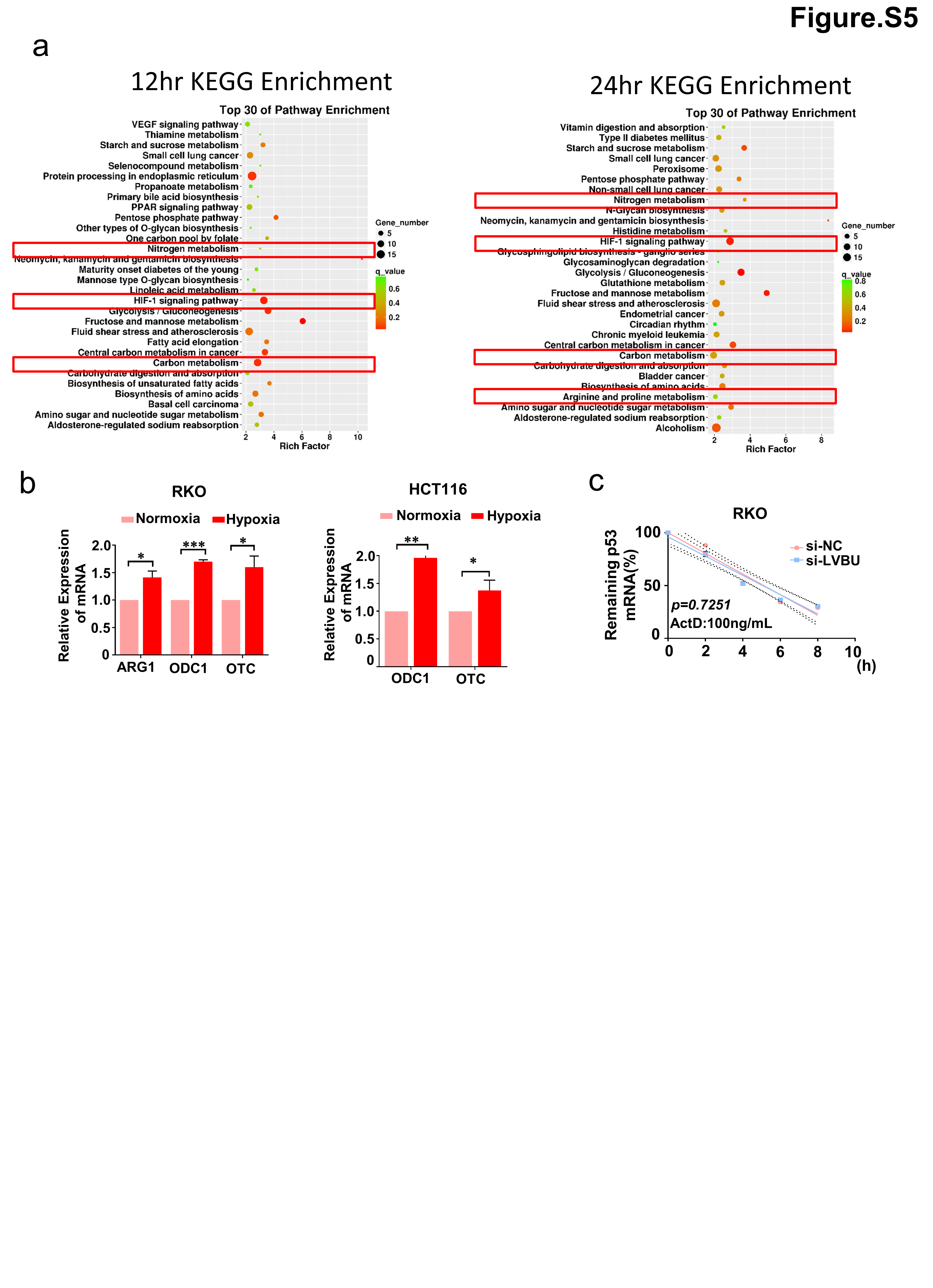

Supplement: Supplementary file 8 — Supplementary Figure 5 [file 41388_2022_2413_MOESM8_ESM.tif]

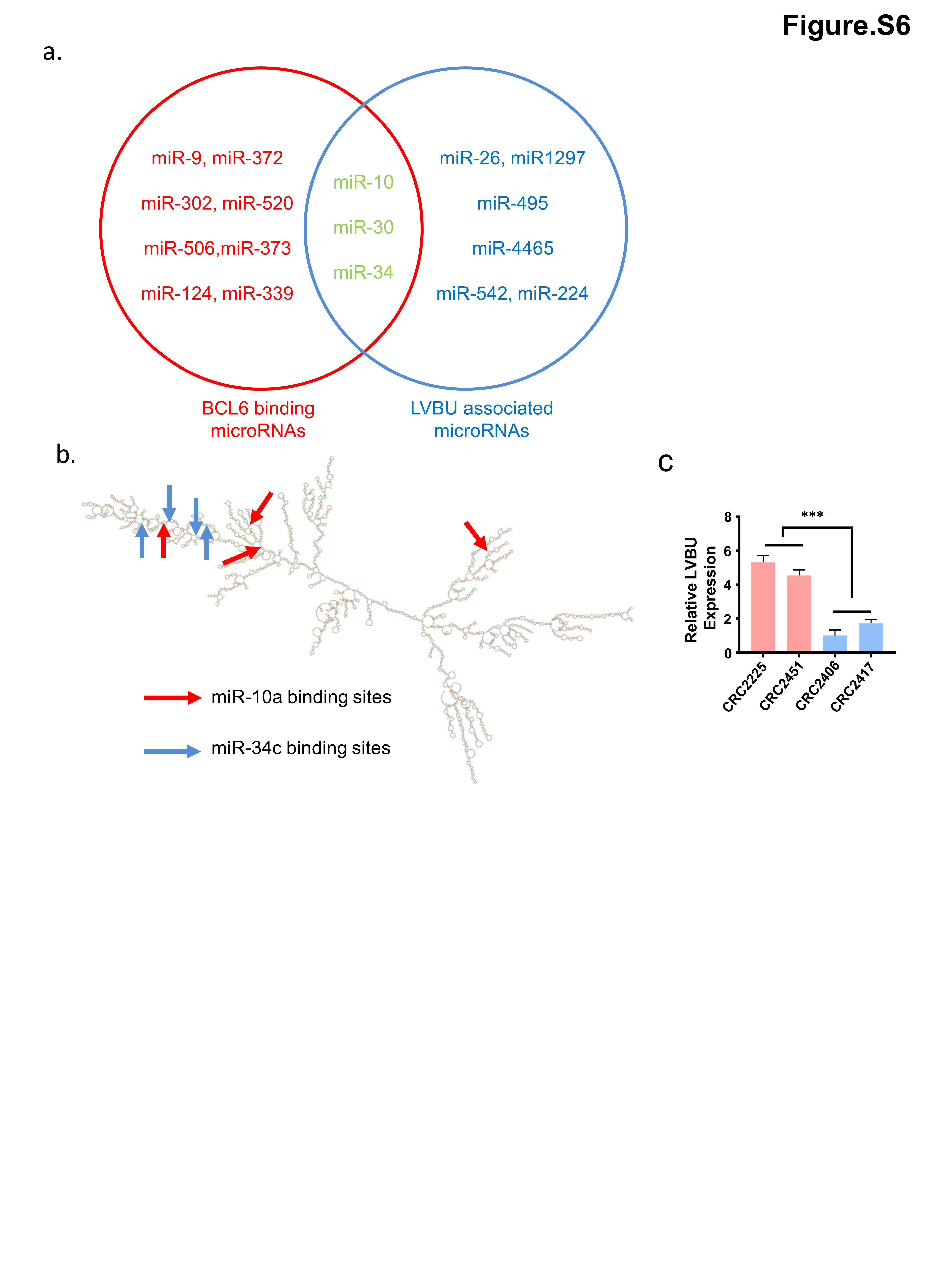

Supplement: Supplementary file 9 — Supplementary Figure 6 [file 41388_2022_2413_MOESM9_ESM.tif]
